# Potential for Sulfate Reduction in Mangrove Forest Soils: Comparison between Two Dominant Species of the Americas

**DOI:** 10.3389/fmicb.2016.01855

**Published:** 2016-11-18

**Authors:** Melike Balk, Joost A. Keuskamp, Hendrikus J. Laanbroek

**Affiliations:** ^1^Department of Microbial Ecology, Netherlands Institute of Ecology–Royal Netherlands Academy of Arts and SciencesWageningen, Netherlands; ^2^Faculty of Geosciences, Utrecht UniversityUtrecht, Netherlands; ^3^Ecology and Biodiversity Group, Department of Biology, Utrecht UniversityUtrecht, Netherlands

**Keywords:** sulfate reduction, mangroves, *Avicennia germinans*, *Rhizophora mangle*, dsrB gene

## Abstract

*Avicennia* and *Rhizophora* are globally occurring mangrove genera with different traits that place them in different parts of the intertidal zone. It is generally accepted that the oxidizing capacity of *Avicennia* roots is larger than that of *Rhizophora* roots, which initiates more reduced conditions in the soil below the latter genus. We hypothesize that the more reduced conditions beneath *Rhizophora* stands lead to more active sulfate-reducing microbial communities compared to *Avicennia* stands. To test this hypothesis, we measured sulfate reduction traits in soil samples collected from neighboring *Avicennia germinans* and *Rhizophora mangle* stands at three different locations in southern Florida. The traits measured were sulfate reduction rates (SRR) in flow-through reactors containing undisturbed soil layers in the absence and presence of easily degradable carbon compounds, copy numbers of the *dsrB* gene, which is specific for sulfate-reducing microorganisms, and numbers of sulfate-reducing cells that are able to grow in liquid medium on a mixture of acetate, propionate and lactate as electron donors. At the tidal locations Port of the Islands and South Hutchinson Islands, steady state SRR, *dsrB* gene copy numbers and numbers of culturable cells were higher at the *A. germinans* than at the *R. mangle* stands, although not significantly for the numbers at Port of the Islands. At the non-tidal location North Hutchinson Island, results are mixed with respect to these sulfate reduction traits. At all locations, the fraction of culturable cells were significantly higher at the *R. mangle* than at the *A. germinans* stands. The dynamics of the initial SRR implied a more *in situ* active sulfate-reducing community at the intertidal *R. mangle* stands. It was concluded that in agreement with our hypothesis *R. mangle* stands accommodate a more active sulfate-reducing community than *A. germinans* stands, but only at the tidal locations. The differences between *R. mangle* and *A. germinans* stands were absent at the non-tidal, impounded location.

## Introduction

Mangrove species inhabiting tropical and subtropical coastal zones are adapted to tidal influences on soil temperature, water content and salt concentration, and to varying degrees of anoxia (Alongi, 2008). Species-specific adaptations of propagule dispersal, salt tolerance physiology, and root zone aeration often lead to zonation of species perpendicular to the shoreline. *Avicennia* and *Rhizophora*, both mangrove genera with a global distribution, developed different mechanisms to adapt to stress factors imposed by the prevailing tidal regime. Due to these differences, *Avicennia* and *Rhizophora* generally form mono-specific stands at distinct positions in the tidal zone. In Florida, for example, *Rhizophora mangle* usually occurs lower in the intertidal zone than *Avicennia germinans* that can be found more at in-land sites where tidal inundation is less frequent (McKee, 1993).

In most regions, mangrove forests are characterized by high primary production (Bouillon et al., 2008; Kristensen et al., 2008). It was estimated that on average 40% of the mangrove production is decomposed in the soil, 30% is exported from the forest, 10% is stored in the soil, and 9% is consumed by herbivores (Duarte and Cebrian, 1996). Hence, a major part of primary mangrove production is decomposed locally. Microbial decomposition will depend on soil environmental conditions, which in the case of mangroves are largely governed by the prevailing tidal regime (Keuskamp et al., 2015). Aerobic microbial processes, which are thermodynamically most favorable (Laanbroek, 1990), are restricted to the first few oxygen-containing mm’s of the soil as has been shown for soils covered by *Avicennia marina* and *Rhizophora apiculata* (Andersen and Kristensen, 1988; Kristensen et al., 1988, 1992, 1994). Through the presence of aerenchyma in specialized root structures, both *Avicennia* spp. and *Rhizophora* spp. are able to transfer oxygen from the air to their roots in anoxic sediments (Scholander et al., 1955) facilitating aerobic decomposition at deeper soil layers. Several observations suggest that *Avicennia* species generally maintain a more oxidized root zone compared to *Rhizophora* species (Nickerson and Thibodeau, 1985; Thibodeau and Nickerson, 1986; Alongi et al., 2000). Since the redox status in the root zone is determined by the balance between oxygen-producing and -consuming processes, the more oxidized zones with *Avicennia* are likely due to a higher oxygen to labile carbon ratio released from the roots of this mangrove species compared to *Rhizophora* species.

Sulfate reduction, which is accomplished by strictly anaerobic microorganisms, is globally the second most important respiratory process after aerobic respiration involved in the decomposition of mangrove-derived soil organic matter (Kristensen et al., 2008). An earlier study by Balk et al. (2015) suggests an effect of depth on traits of the sulfate-reducing microbial community in mangrove forest soils. Potential sulfate reduction rates, copy numbers of the *dsrB* gene, which is specific for sulfate-reducing microbes, and numbers of sulfate-reducing cells that were able to grow on a mixture of acetate, propionate and lactate were all significantly higher in samples from the reduced sub-surface layers (4–6 cm depth) than in samples from the more oxidized surface layers (0–2 cm depth). Hence, traits of the sulfate-reducing community are strongly influenced by the redox status of the soil. Assuming more reduced conditions below *Rhizophora* than below *Avicennia* stands, we hypothesize that the soil from beneath *Rhizophora* has higher potential sulfate reduction rates and higher numbers of sulfate-reducing microorganisms than soil from underneath *Avicennia*. To test this hypothesis, we collected the upper 2 cm of soil from beneath *A. germinans* and *R. mangle* stands on three locations in South Florida that differ mutually in tidal regimes. At each location, both species were present in separated zones. Since oxygen penetrates generally only a few mm’s into the soil (Andersen and Kristensen, 1988; Kristensen et al., 1988, 1992, 1994), we assumed that differences among species in the upper 2 cm of the soil can largely be ascribed to differences in the balance between oxygen and carbon released by the plant roots.

## Materials and Methods

### Study Sites

In March 2009, soil samples were collected from monospecific stands of *Avicennia germinans* and *Rhizophora mangle* at three mangrove forest locations in southern Florida that differ mutually in total organic carbon content (**Table 1**). One sampling location was situated at the Port of the Islands (25°56′ N and 81°30′ W) in Collier County on the east coast of the Gulf of Mexico, one location near Jensen Beach (27°17′ N and 80°13′ W) in Martin County on South Hutchinson Island, and one location near Fort Pierce (27°28′N and 80°18′W) in St. Lucie County on North Hutchinson Island. North and South Hutchinson Islands are part of a range of barrier islands sheltering the Indian River lagoon from the North Atlantic Ocean. Both locations are situated on the land side of the islands along the lagoon. At the moment of sampling, the mangrove forest at North Hutchinson Island was impounded and tidal influences were restricted (Verhoeven et al., 2014). The absence of tide at this location affected mostly the stands of *A. germinans*. At the tidal locations Port of the Islands and South Hutchinson Island, intertidal soils from *R. mangle* stands were collected from the seaward fringes, while supratidal soils from *A. germinans* were sampled more from the interior of the forests. In all cases, soils were collected from mature stands with an average tree height of more than 2 m. The soil samples were taken close to the aerial roots of the trees. Replicate samples from 0 to 2 cm depth were collected within an area of 1 m^2^ and transported to the laboratory on ice.

**Table 1:** Characteristics of mangrove forest soil samples collected from Florida, USA.

Mangrove species	*Avicennia germinans*			*Rhizophora mangle*		
Location	Port of the Islands	South Hutchinson Island	North Hutchinson Island	Port of the Islands	South Hutchinson Island	North Hutchinson Island
Pore water salinity (g/L)	69.0	41.0	94.5	38.5	37.0	57.0
Pore water pH	7.7	5.7	7.5	7.4	7.4	6.7
Mean particle size (DV50^a^)	83.9	91.5	156.7	86.5	115.8	291.7
Total organic carbon (% dw)	26.2	26.0	3.5	22.3	13.0	6.1

^a^*As volume-based mean diameter.*

### Determination of Sulfate Reduction Rates

Sulfate reduction rates were determined in the flow-through reactors (FTRs) as described before (Balk et al., 2015). Shortly, the incubation of intact soil layers ran in these reactors at a constant rate of 1 ml h^-1^ in a temperature-controlled room at 20–22°C. To maintain anoxic conditions throughout the experiments, inflow solutions and connecting tubing were purged with argon and the incubations were performed in the dark to avoid oxygen production by photosynthesis. Before the start of the incubation experiments, reactors were flushed with a salt solution containing various concentrations of NaCl that match the salt concentrations measured at the sampling sites (**Table 1**). Flushing with salt solutions made it also possible to establish the time it takes a solution to move from the bottom to the top of the soil in the reactor. These so-called breakthrough times for Port of the Islands, South Hutchinson Island and North Hutchinson Island were 65, 61, and 52 h for the *Avicennia* soils, respectively, and 69, 58, and 52 h for the corresponding *Rhizophora* soils. These breakthrough times correlated well (*R*^2^ = 0.765) with the total carbon contents presented in **Table 1**. During the first 90 h of the incubation experiment, the inflow solutions contained 4 mM sodium sulfate, defined concentrations of NaCl and no electron donor. In order to determine the effect of easily available organic carbon on sulfate reduction rates, both sodium acetate (10 mM) and sodium lactate (10 mM) were supplied after 90 h, while sodium sulfate was increased to 8 mM to fulfill the demand for extra oxidant capacity. Sulfate concentrations were measured by ion chromatography using a Dionex DX120 (Water, Milford, MA, USA) with an IonPac ICE-AS6 column and Anion-ICE Micro Membrane II suppressor. Steady-state sulfate reduction rates (SRR) were calculated as described before (Balk et al., 2015).

### DNA Extraction

Chromosomal DNA was isolated from the soils by a modification of the DNA isolation procedure as described previously by Zhou et al. (1996). DNA was further purified using the DNA Clean & Concentrator kit (Zymo Research, Orange, CA, USA). The quantity and quality of the extracted DNA were analyzed by spectrophotometry using a ND-1000 spectrophotometer (NanoDrop Technologies, Wilmington, DE, USA) and by agarose gel electrophoresis. The genomic DNA was stored at -20°C for further analyses.

### qPCR Amplification

qPCR amplification for sulfate-reducing prokaryotes targeting on the *dsrB* gene was performed in a total volume of 20 μl with the primer pair DSRp2060F and DSR4R (Geets et al., 2006) on a Rotor-Gene 3000 (Corbett Research, QIAGEN, Valencia, CA, USA). Each PCR mixture was made using a CAS-1200 pipetting robot (Corbett Research, QIAGEN, Valencia, CA, USA) and contained 3 μl diluted template, 10 μl Absolute^TM^ QPCR SYBR Green Mix (Thermo Scientific, Epsom, UK), 0.4 μl of each primer (10 μM) and 1 μl Bovine Serum Albumin (BSA; 20 mM). The qPCR procedure was as follows: 10 s at 95°C for initial denaturation, 45 cycles of 20 s at 95°C, 30 s at 56°C, and 45 s at 72°C. At each cycle, fluorescence was obtained at 84°C. A melting curve was performed from 55°C to 99°C to confirm PCR product specificity. Purified PCR products from extracted DNA originating from the same soil samples, generated with the same primer set and cloned into the pGEM-T Easy Vector (Promega, Madison, WI, USA) were used for the production of the standard curve. In order to get specific products and avoid inhibition, dilution series were made of the soil DNA solution to test for inhibition and set a 100-fold dilution as the final template. The amplification efficiency ranged from 98 to 104% with *R*^2^ values greater than 0.99.

### Enumeration of Sulfate-Reducing Microorganisms Able to Grow on a Mixture of Acetate, Propionate, and Lactate

A Most Probable Number (MPN) assay was applied for enumeration of sulfate-reducing microorganisms able to grow on a mixture of acetate, propionate and lactate (APL medium). Briefly, soil was re-suspended in phosphate-buffered saline (pH 7.4) in a soil to buffer ratio of 1:6 and shaken for 2 h. Subsequently, 10-fold dilutions were made in microtiter plates containing a minimal salt medium (Widdel and Bak, 1992) supplemented with a mix of acetate, propionate and lactate (15 mM each) as electron donors. Sodium sulfate (20 mM) was provided as the electron acceptor. Sodium thioglycolate (0.5 mM) was added as reducing agent and iron sulfate (0.2 mM) as indicator for the occurrence of sulfate reduction. The formation of black iron sulfide precipitates was indicative for sulfate reduction. The microtiter plates were incubated for 3 months at 25°C in anaerobic incubation bags (Anaerocult^®^ A mini, Merck, Darmstadt, Germany). Based on the number of positive wells, the most probable numbers and related confidence limits were calculated using standard tables (Rowe et al., 1977).

### Statistics

The obtained data were fitted to (mixed) linear models in R 3.2.3 (R Core Team, 2015) with site as a random factor where appropriate. Normality and homoscedasticity of residuals were visually assessed and confirmed by Shapiro–Wilk and Levene’s tests, respectively. Where necessary, log transformations were applied prior to analysis. Overall effects were tested for significance using ANOVA with type II sum of squares using *car* 2.0-22 (Fox and Weisberg, 2011), while pairwise comparisons were conducted using Tukey’s HSD as implemented in the *multcomp* 1.4-6 package (Hothorn et al., 2008). Due to the commonly observed interactive effect of plant species and locations, the effects of both fixed variables on the sulfate reduction traits were tested separately.

## Results

### Potential Sulfate Reduction Rates

**FIGURE 1:**
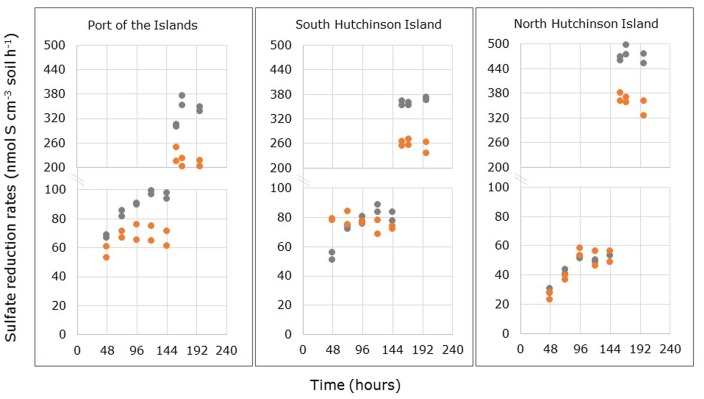
**Sulfate reduction rates (SRR) measured in through-flow reactors containing the top 2 cm of intact soil samples collected from *Avicennia germinans* (gray dots) and *Rhizophora mangle* stands (orange dots) at Port of the Islands, South Hutchinson Island and North Hutchinson Island.** Note the split *Y*-axes for the points presented after 144 h.

Differences between sulfate concentrations in- and outflowing the FTRs filled with undisturbed mangrove soils reflect the activity of sulfate-reducing microorganisms in these soils. **Figure 1** presents the SRR measured in the FTRs. The increase in rates after 144 h was due to the amendment of easily degradable carbon sources (i.e., acetate and lactate) to the inflowing medium. These carbon sources were added 90 h after the start of the incubation. Delay between amendment and observation of its effects was determined by the breakthrough time, which is the time needed for the solution to pass the entire reactor. In the non-amended incubations of soils samples from Port of the Islands and South Hutchinson Island, the reactors reached steady state SRR within 96 and 48 h, respectively, for *R. mangle* soils, while soils from *A. germinans* required 120 h to reach steady state SRR. Such a difference between *A. germinans* or *R. mangle* stands was not observed in North Hutchinson Island: Soils from both species required 96 h to reach steady state rates.

For the calculation of average steady state SRR two periods were used: One from 96 to 144 h measured in the absence of added carbon sources, and one from 160 to 198 h determined in the presence of added acetate plus lactate. The results are presented in **Table 2**. When only the natural carbon sources were available, steady state SRR were significantly higher in soil samples from *A. germinans* stands than in soil samples from *R. mangle* stands for the locations Port of the Islands and South Hutchinson Island (*p* < 0.001 and *p* < 0.01, respectively), while in samples from North Hutchinson Island no significant differences were observed between stands from both plant species (Supplementary Table S1). Sampling location had a significant effect on measured steady state SRR (*p* < 0.001). Due to interactive effects of plant species and sampling locations, the effect of location on steady state SRR was tested separately for each species (Supplementary Table S2). In *R. mangle* stands, no significant differences in SRR were observed between Port of the Islands and South Hutchinson Island, although both showed significantly higher steady state rates compared to soil from North Hutchinson Island. In soils from *A. germinans* stands, steady state SRR in the absence of added organic compounds differed significantly between locations: North Hutchinson Island < South Hutchinson Island < Port of the Islands. Hence with both mangrove species, steady state SRR were significantly lower in samples from North Hutchinson Island.

**Table 2:** Steady-state sulfate reduction rates (SRR) (nMol S cm^-3^ h^-1^) measured in non-carbon-amended and in carbon-amended flow-through reactors (FTRs) containing the upper 2 cm of soils collected from *Avicennia germinans* or *Rhizophora mangle* stands.

	Non-carbon amended reduction rates			Carbon-amended reduction rates		
	Port of the Islands	South Hutchinson Island	North Hutchinson Island	Port of the Islands	South Hutchinson Island	North Hutchinson Island
*Avicennia germinans*	97	83	49	336	361	471
*Rhizophora mangle*	68	73	52	217	257	359

**FIGURE 2:**
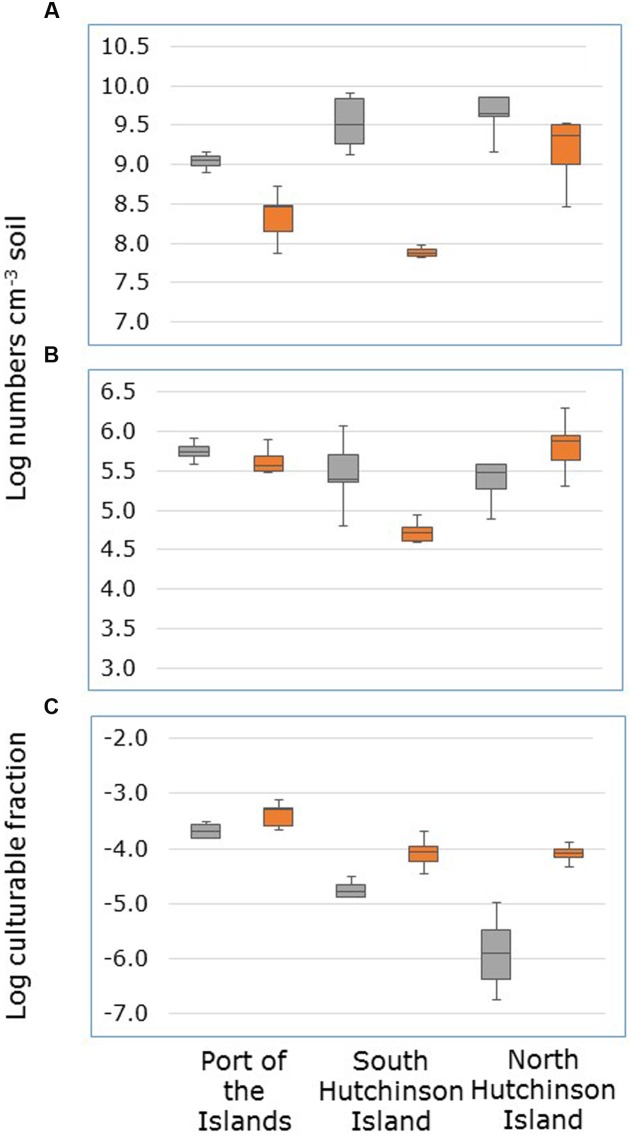
**Numbers of *dsrB* gene copies (A)** numbers of sulfate-reducing cells able to grow on liquid medium with acetate, propionate, lactate (APL medium) **(B)** and the fraction of *dsrB* gene-containing cells able to grow on APL medium **(C)** in soil samples collected from *Avicennia germinans* (gray boxplots) and *Rhizophora mangle stands* (orange boxplots) at Port of the Islands, South Hutchinson Island and North Hutchinson Island.

The rate differences between *A. germinans-* and *R. mangle*-derived soils became more pronounced by addition of extra electron donors (**Figure 2**; **Table 2**). Steady state SRR were consistently higher in soil samples from *A. germinans* stands than in samples from *R. mangle* stands (Supplementary Table S1). Sampling location had also a significant effect on these rates (*p* < 0.001). For soils from *R. mangle* and *A. germinans* stands, steady state SRR increased significantly between locations in the same order: Port of the Islands < South Hutchinson Island < North Hutchinson Island (Supplementary Table S2).

### Numbers of Sulfate-Reducing Microorganisms

Numbers of sulfate-reducing microorganisms in the mangrove soil were enumerated in two ways, i.e., by determining the copy numbers of the *dsrB* gene and by establishing the number of cells able to grow in APL medium by an MPN method. Numbers of these latter cells were on average 96000 times lower than the copy numbers of the *dsrB* gene.

Copy numbers of the *dsrB* gene were on average higher in soil samples from *A. germinans* stands than from *R. mangle* stands (**Figure 2A**). However, this was only significant for samples collected at South Hutchinson Island (Supplementary Table S1). Due to the interactive effects of plant species and sampling locations, the effect of sampling location on *dsrB* gene copy numbers was tested separately for both mangrove species. For *R. mangle* stands, gene copy numbers were significantly higher in samples from North Hutchinson Island than in samples from Port of the Islands and South Hutchinson Island that did not significantly differ from each other (Supplementary Table 2). For *A. germinans* stands, gene copy numbers were significantly lower in samples from Port of the Islands than in samples from South and North Hutchinson Island that did not significantly differ from each other.

The effect of numbers of cells that grew in APL medium was also dependent on sampling location (**Figure 2B**). Only in soil samples from South Hutchinson Island, significantly higher cell numbers were observed in samples from *A. germinans* stands than in samples from *R. mangle* stands (Supplementary Table S1). At the other locations, numbers of cells able to grow on a mixture of acetate, propionate, and lactate did not differ significantly between *A. germinans* and *R. mangle* stands. For *A. germinans* stands sampling location had no significant effect on the number of such cells, but for *R. mangle* stands these numbers were significantly higher at North Hutchinson Island than at Port of the Islands and South Hutchinson Island, which did not differ significantly from each other (Supplementary Table S2).

Both plant species and sampling location had a significant effect on the fraction of *dsrB*-containing cells that were able to grow in APL medium (Supplementary Table S3). This fraction was higher in the *R. mangle* stand than in the *A. germinans* stands, and higher at Port of the Islands than at the other locations (**Figure 2C**).

## Discussion

Based on the generally accepted idea that *Rhizophora* soils are more reduced than *Avicennia* soils, we hypothesized that sulfate reduction traits such as community size and activity would be more manifest in soils from *Rhizophora* stands than in soils from *Avicennia* stands. A number of observations seemed to disprove the hypothesis. Potential steady state reduction rates, copy numbers of the *dsrB* gene, and numbers of culturable cells were all significantly higher values in samples obtained from *A. germinans* stands than in samples from *R. mangle* stands, at least at the tidal locations Port of the Islands and South Hutchinson Island. Then the question arises what causes the unexpected findings of higher sulfate reduction traits in *A. germinans* stands than in *R. mangle* stands, which are assumed to be more reduced than the *Avicennia* locations?

Was the original assumption correct that *Avicennia* is able to maintain a more oxidized root zone than *Rhizophora*? By a series of gas exchange experiments Scholander et al. (1955) showed that both *Avicennia nitida* (synonymous with *A. germinans*) and *R. mangle* are able to supply their aerenchymatous roots with oxygen from the atmosphere, albeit with different temporal cycles. Both *A. nitida* and *R. mangle* showed diurnal cycles with the highest oxygen concentrations at day time. However, *A. nitida* growing in the tidal zone revealed also tidal cycles with the highest oxygen concentrations during low tide. Thibodeau and Nickerson (1986) measured a decreasing redox potential in the soil with an increasing distance to *A. germinans* trees, whereas the opposite, i.e., increasing redox potential with increasing distance to the trees, was observed with *R. mangle*. As shown in another publication by Nickerson and Thibodeau (1985), the root zone-oxidizing capacity of *A. germinans* may lead to growth of this species in more sulfide-rich soils than *R. mangle*. In contrast to *A. germinans*, *R. mangle* is not able to lower the sulfide concentration of the surrounding soil. In a study on below-ground decomposition of organic matter in mangrove forest dominated by *Avicennia marina* or *Rhizophora stylosa*, Alongi et al. (2000) observed lower redox potential in the upper soil layers of *R. stylosa* stands than in the upper soil layers of *A. marina* stands, even when both species grew in the same tidal zone. In only one case studied by Alongi et al. (2000), a lower redox potential was observed in the upper layers of an *A. marina* stand. The authors suggested that such a difference in behavior between the species may be due to differential exposure to wave action and perhaps to differences in flooding and drainage cycles. So in general, we have no reason to assume that our *R. mangle* stands are less reduced than our *A. germinans* stands. The more so because the intertidal *R. mangle* stands at the tidal locations Port of the Islands and South Hutchinson Island were flooded twice daily, whereas the supratidal *A. germinans* stands were flooded seldom. With respect to the non-tidal stands in the impounded mangrove forest of North Hutchinson Island, the *R. mangle* stands were always wetter than the *A. germinans* stands. Another indication of more oxidized conditions at the *A. germinans* stands in our study may the higher salinities measured pair-wise in the *A. germinans* and *R. mangle* stands at the three locations. A higher salinity reflects a higher degree of evapotranspiration and consequently a higher degree of air drawn into the soil.

Might the lower *dsrB* gene copy numbers observed at the tidal *R. mangle* stands be explained by inhibition of the qPCR procedure by the presence of inhibiting compounds? A generally low nutrient content combined with a relatively high content of condensed tannins and phenolic compounds, lead generally to a lower carbon decomposition rate and consequently to more organic carbon accumulation at *Rhizophora* stands than at *Avicennia* stands (Robertson, 1988; McKee and Faulkner, 2000; Middleton and McKee, 2001; Erickson et al., 2004; Kristensen et al., 2008; Galeano et al., 2010; Lima and Colpo, 2014). The *A. germinans* and *R. mangle* stands have been selected pair-wise at three locations in mutually remote regions. These pair-wise stands showed always a higher total carbon content at the *A. germinans* than at the *R. mangle* stands, except at the impounded, non-tidal location of North Hutchinson. However, we cannot exclude that certain carbon compounds specifically present at *R. mangle* stands have affected the enumeration of *dsrB* gene copies by qPCR negatively. An argument against a possible inhibition of the qPCR enumeration by organic compounds present at the *R. mangle* stands, might be the distribution of culturable cell numbers at the different locations that show the same trends as observed with *dsrB* gene copies. Finally, the trend of higher numbers at *A. germinans* stands compared to *R. mangle* stands, reflects the higher potential SRR measured in samples from *A. germinans* stands.

Were the levels of *dsrB* gene copy numbers very different form numbers determined in other studies? Irrespective of *A. germinans* or *R. mangle*, the median abundance of the *dsrB* gene we observed at the different stands varied between 7.5 × 10^7^ and 4.4 × 10^9^ copies per cm^3^, which is slightly above the range we found in the carbon-poor *A. marina* soils at the Red Sea coast (Balk et al., 2015). At the upper 5 cm of an oil-contaminated, Brazilian mangrove soil, Andrade et al. (2012) observed 3.6 × 10^8^ *dsrB* gene copies per g soil (Andrade et al., 2012). In other contaminated mangrove soils in Brazil, the *dsrB* gene copy numbers varied from 7.9 × 10^4^ to 2.0 × 10^5^ per g soil (Varon-Lopez et al., 2014). These last numbers are lower than the other numbers from mangrove soils, but Varon-Lopez et al. (2012) mixed the upper 30 cm of the soil before they started their analyses. From the work of Andrade et al. (2012) it is known that *dsrB* gene copy numbers decline with depth. The numbers of *dsrB* gene copies found in our study were at the same range as found for example in estuarine sediments (Kondo et al., 2004) and in paddy soil (Liu et al., 2009).

A striking observation was the significantly larger fraction of culturable cells in samples from the *R. mangle* stands than in samples from the *A. germinans* stands, although absolute numbers of *dsrB*-containing and growing cells were larger at the *A. germinans* stands. A larger fraction of culturable cells might be explained by differences in community composition, but this was not determined in this study. In our former study on sulfate-reducing communities at the Red Sea coast we found a higher fraction of culturable cells in the deeper soil layers than in the surface layers, but we were not able to relate this to differences in genus diversity and to differences in functional diversity (Balk et al., 2015). So most likely, the higher fraction of culturable cells mimicked more active sulfate-reducing communities at the intertidal *R. mangle* stands.

The presence of a more active sulfate-reducing community present at intertidal *R. mangle* stands than at supratidal *A. germinans* stands may also be inferred from the differences in time required to obtain steady state rates as was observed during the measurements of potential sulfate-reducing activities. At the tidal locations, samples from *R. mangle* stands collected at Port of the Islands and South Hutchinson Island required 96 and 48 h, respectively, before steady state rates were obtained, while the corresponding time for samples from *A. germinans* stands amounted to 120 h for both locations. No difference with respect to reaching steady state SRR was observed between *R. mangle* and *A. germinans* samples collected at the impounded, non-tidal location of North Hutchinson Island. The time required to reactivate the cells from the more oxidized stands may be due to the establishment of anoxic conditions upon starting the measurements in the FTR’s. Sulfate-reducing microorganisms have been observed in oxic parts of estuarine and lake sediments before (Laanbroek and Pfennig, 1981; Sass et al., 1997). Sulfate-reducing strains isolated from oxic sediment layers revealed a higher oxygen tolerance and capacity of oxygen respiration than strains originating from anoxic sediment layers (Sass et al., 1997). In this latter study, no sulfate reduction was observed under oxic conditions because oxygen was preferentially reduced. All strains of a diverse group of sulfate-reducing isolates were able to oxidize lactate and even to oxidize sulfide in the presence of oxygen thereby creating again anoxic conditions (Sass et al., 2002).

The impounded, non-tidal location at North Hutchinson Island reacted also more strong than the tidal stations to the addition of acetate and lactate as electron donors to the FTR’s used for determining potential SRR. This may be explained by a different community present in the non-tidal stands or on repression of sulfate reduction by organic compounds present in the tidal stands. With respect to this latter explanation: The supratidal *A. germinans* stands at the tidal locations showed a more intense effect in the FTR’s upon the addition of acetate and lactate than the intertidal *R. mangle* stands, while these tidal stands contained more organic carbon than the samples from the non-tidal North Hutchinson Island location. Hence, it seems that more oxidized habitats at the non-tidal stations and at the supratidal *A. germinans* stands at the tidal stations contain a community able to react more intense of the addition of acetate and lactate once they have been activated by creating anoxic conditions in the reactors.

## Conclusion

Firstly, when potential sulfate-reduction rates and total numbers of *dsrB* gene copies and of culturable cells are not the most reliable indicators for active sulfate-reducing communities, but when the dynamics of SRR measured during the initial period in the FTR’s and the size of the fraction of culturable cells are, then the tidal stands of *R. mangle* accommodate a more active sulfate-reducing community than the stands of *A. germinans*, and we cannot disprove our hypothesis.

Secondly, the supratidal *A. germinans* stands at the tidal locations and the *A. germinans* and *R. mangle* stands at the impounded, non-tidal location accommodate apparently a sulfate-reducing community that is able to survive oxidized conditions.

## Author Contributions

MB collected the samples, performed the analyses, took part in the discussions and participated in writing of the manuscript. JK performed the statistical analyses, took part in the discussions and participated in writing of the manuscript. HL collected the samples, took part in the discussions and participated in writing of the manuscript.

## Conflict of Interest Statement

The authors declare that the research was conducted in the absence of any commercial or financial relationships that could be construed as a potential conflict of interest.
