# Supplementary material for: Potential for Sulfate Reduction in Mangrove Forest Soils: Comparison between Two Dominant Species of the Americas
Source: Front Microbiol. 2016 Nov 18;7:1855. doi: 10.3389/fmicb.2016.01855 (PMC5114281; doi:10.3389/fmicb.2016.01855)
Supplement: Supplementary file 1 [file Table_1.PDF]

Supplementary Table S1: Results of a mixed linear model of the effect of dominant mangrove species on a number of traits related to sulfate reduction. For each combination of location and variable, the null hypothesis has been listed, followed by an estimate of mean and standard error based on a restricted maximum likelihood (REML) procedure. In addition, the test statistic and estimated p value are displayed.

**Dependent variable**	**Location**	**Hypothesis**	**Estimate**	**SE**	**χ^2 / F[a]**	**Df**	**P[b]**
Non-C-amended maximum sulfate reduction rates	Port of the Islands	*R. mangle - A. germinans* = 0	-25.633	2.965	74.761	1	<2.20E-16***
	South Hutchinson Island	*R. mangle - A. germinans* = 0	-7.203	2.409	8.9447	1	0.0028**
	North Hutchinson Island	*R. mangle - A. germinans* = 0	2.088	3.921	0.2836	1	0.5944
C-amended maximum sulfate reduction rates	Port of the Islands	*R. mangle - A. germinans* = 0	-119.38	13.75	75.357	1	<2.20E-16***
	South Hutchinson Island	*R. mangle - A. germinans* = 0	-103.488	8.612	144.42	1	<2.20E-16***
	North Hutchinson Island	*R. mangle - A. germinans* = 0	-112.47	14.76	58.03	1	2.58E-14***
dsrB gene copy numbers	Port of the Islands	*R. mangle - A. germinans* = 0	-1.1785	0.5516	4.5644	1	0.0651
	South Hutchinson Island	*R. mangle - A. germinans* = 0	-3.516	0.447	61.894	1	4.925e-05 ***
	North Hutchinson Island	*R. mangle - A. germinans* = 0	-1.0497	0.5491	3.654	1	0.0923
Culturable cell numbers	Port of the Islands	*R. mangle - A. germinans* = 0	-0.6285	0.3979	2.4951	1	0.1529
	South Hutchinson Island	*R. mangle - A. germinans* = 0	-1.705	0.5085	11.241	1	0.0100 **
	North Hutchinson Island	*R. mangle - A. germinans* = 0	0.5562	0.6868	0.6557	1	0.4415

[a] Sulfate reduction rates were tested using the χ^2-distribution, while gene copy and culturable cell numbers were tested using the F-distribution;
[b] Significance codes: *** 0.001, ** 0.01
