# Supplementary material for: Potential for Sulfate Reduction in Mangrove Forest Soils: Comparison between Two Dominant Species of the Americas
Source: Front Microbiol. 2016 Nov 18;7:1855. doi: 10.3389/fmicb.2016.01855 (PMC5114281; doi:10.3389/fmicb.2016.01855)
Supplement: Supplementary file 2 [file Table_2.PDF]

Supplementary Table S2: Results of a mixed linear model of the effect of dominant mangrove species on a number of traits related to sulfate reduction. For each combination of location and variable, the null hypothesis has been listed, followed by an estimate of mean and standard error based on a restricted maximum likelihood (REML) procedure. In addition the z value and resulting p value based on a post-hoc Tukey HSD are displayed. POI = Port of the Islands, SHI = South Hutchinson Island, NHI = North Hutchinson Island

**Dependent variable**	**Species**	**Hypothesis**	**Estimate[a]**	**SE**	**z**	**P r(>|z|)[b]**
Non-C-amended maximum sulfate reduction rates	*R. mangle*	POI - NHI = 0	15.652	3.802	4.117	<1e-04***
		SHI - NHI = 0	21.182	3.802	5.572	<1e-04***
		SHI – POI = 0	5.530	3.802	1.455	0.1460
	A. germinans	POI - NHI = 0	43.204	2.301	18.780	<1e-08***
		SHI - NHI = 0	30.304	2.301	13.170	<1e-08***
		SHI – POI = 0	-12.900	2.194	-5.880	<1e-08***
C-amended maximum sulfate reduction rates	*R. mangle*	POI - NHI = 0	-142.055	12.340	-11.509	<0.001***
		SHI - NHI = 0	-101.325	12.340	-8.209	<0.001***
		SHI – POI = 0	40.730	12.340	3.300	<0.001***
	A. germinans	POI - NHI = 0	-135.145	2.194	-9.856	<1e-04***
		SHI - NHI = 0	-110.312	11.190	2.219	<1e-04***
		SHI – POI = 0	24.833	11.190	18.78	0.0265*
dsrB gene copy numbers	*R. mangle*	POI - NHI = 0	-1.9085	0.5222	-3.655	0.0003 ***
		SHI - NHI = 0	-2.6872	0.5222	-5.146	< 1e-04 ***
		SHI – POI = 0	-0.7787	0.5222	-1.491	0.1359
	A. germinans	POI - NHI = 0	-1.7797	0.5142	-3.461	0.0016 **
		SHI - NHI = 0	0.2205	0.5142	-0.429	0.6680
		SHI – POI = 0	1.5592	0.5142	3.032	0.0024 **
Culturable cell numbers	*R. mangle*	POI - SHI = 0	-0.7836	0.4555	-1.720	0.0854
		SHI - NHI = 0	-2.5066	0.4555	-5.503	< 1e-04 ***
		POI - NHI = 0	-1.7230	0.4555	-3.783	0.0002 ***
	A. germinans	No significant effect of sampling location on culturable cell numbers				

[a] Estimates are made for Ln-transformed *dsrB* gene copy numbers and numbers of viable cells; [b] significance codes: *** 0.001, ** 0.01
