# Supplementary material for: Potential for Sulfate Reduction in Mangrove Forest Soils: Comparison between Two Dominant Species of the Americas
Source: Front Microbiol. 2016 Nov 18;7:1855. doi: 10.3389/fmicb.2016.01855 (PMC5114281; doi:10.3389/fmicb.2016.01855)
Supplement: Supplementary file 3 [file Table_3.PDF]

Supplementary Table S3: Linear model results of the fraction of cells containing the *dsrB* gene (assuming one gene per cell) that are able to grow on a mixture of acetate, propionate and lactate as electron donors. The p values were calculated using type II SS.

**Independent variable**	**F**	**Df**	**P r(>F)[a]**
Location	12.0866	2	0.007488 **
Species	9.1310	1	0.005893 **
Location:Species	1.5849	2	0.464222

NOTE: residuals are slightly-non-normal (Shapiro: 0.021*); [a] significance codes: ** 0.01
